# Modelling blood flow in patients with heart valve disease using deep learning: A computationally efficient method to expand diagnostic capabilities in clinical routine

**DOI:** 10.3389/fcvm.2023.1136935

**Published:** 2023-03-03

**Authors:** Pavlo Yevtushenko, Leonid Goubergrits, Benedikt Franke, Titus Kuehne, Marie Schafstedde

**Affiliations:** ^1^Deutsches Herzzentrum der Charité (DHZC), Institute of Computer-assisted Cardiovascular Medicine, Berlin, Germany; ^2^Institute for Imaging Science and Computational Modelling in Cardiovascular Medicine, Charité – Universitätsmedizin Berlin, Berlin, Germany; ^3^Einstein Center Digital Future, Berlin, Germany; ^4^Berlin Institute of Health, Berlin, Germany

**Keywords:** deep learning, computational fluid dynamics, heart valve disease, aortic stenosis, in-silico modelling, artificial neural network, image-based modelling

## Abstract

**Introduction:**

The computational modelling of blood flow is known to provide vital hemodynamic parameters for diagnosis and treatment-support for patients with valvular heart disease. However, most diagnosis/treatment-support solutions based on flow modelling proposed utilize time- and resource-intensive computational fluid dynamics (CFD) and are therefore difficult to implement into clinical practice. In contrast, deep learning (DL) algorithms provide results quickly with little need for computational power. Thus, modelling blood flow with DL instead of CFD may substantially enhances the usability of flow modelling-based diagnosis/treatment support in clinical routine. In this study, we propose a DL-based approach to compute pressure and wall-shear-stress (WSS) in the aorta and aortic valve of patients with aortic stenosis (AS).

**Methods:**

A total of 103 individual surface models of the aorta and aortic valve were constructed from computed tomography data of AS patients. Based on these surface models, a total of 267 patient-specific, steady-state CFD simulations of aortic flow under various flow rates were performed. Using this simulation data, an artificial neural network (ANN) was trained to compute spatially resolved pressure and WSS using a centerline-based representation. An unseen test subset of 23 cases was used to compare both methods.

**Results:**

ANN and CFD-based computations agreed well with a median relative difference between both methods of 6.0% for pressure and 4.9% for wall-shear-stress. Demonstrating the ability of DL to compute clinically relevant hemodynamic parameters for AS patients, this work presents a possible solution to facilitate the introduction of modelling-based treatment support into clinical practice.

## 1. Introduction

In the medical health sector, the impact of artificial intelligence (AI)-based technologies is steadily increasing. While automated medical image analysis is arguably the most successful domain of medical AI applications (1–4), its use becomes conceivable in almost all medical fields, such as diagnostic assessments (5), prediction of patients prognoses (6, 7), assistance in surgical interventions (8) and many more.

On the other hand, hemodynamic modelling, i.e., the computational modelling of blood flow using computational fluid dynamics (CFD) simulations, is also gaining more and more attention as the availability of computational power steadily increases. Having the potential to improve, facilitate and complement current diagnostic and therapy decision-making processes (9–14), CFD simulations are increasingly applied in cardiovascular research (15–17). In the case of aortic valve disease specifically, CFD simulations are used to predict hemodynamic parameters which may be critical to clinical outcome. These include, but are not limited to, paravalvular leakage (18–20), pressure and wall-shear-stress (WSS) in the aorta after aortic valve replacement for different valve types and sizes (21, 22) as well flow patterns generated by different valve diseases and prosthesis types (15, 23).

However, translation of these models into clinical routine remains cumbersome. Not only are CFD simulations very demanding with respect to time and computational costs (several hours on high-end workstations), they also often require expertise in both engineering and medicine. As a result, diagnosis and/or treatment support solutions based on cardiovascular modelling rarely find their way into clinical practice (24–26). Although there are examples of a successful translation [e.g., HeartFlow® (14)], there is a strong discrepancy between the amount of proposed cardiovascular modelling applications and actual clinical applications.

In this regard, deep learning (DL)-based algorithms are potentially suited to overcome the problem of computational demand for higher order cardiovascular modelling. Once trained, a DL algorithm can provide results quickly with little need for computational power or user experience. Considering that clinicians rarely have access to high-end workstations on the one hand and need to perform diagnosis and treatment planning quickly on the other, the above mentioned advantages of a DL-based method may substantially increase the clinical feasibility of hemodynamic modelling. Several studies already showcased the potential of DL-based methods to accurately predict hemodynamic parameters. Examples of application include coronary arteries (27), aortic coarctation (28) and abdominal aortic aneurysms (29). In this study, we investigate the ability of DL-based methods to model hemodynamics of patients with aortic valve stenosis (AS).

AS represents the leading valvular heart disease with surgical (aortic valve reconstruction or replacement) and interventional (transcatheter aortic valve implantation, TAVI) treatment options that are increasingly applied also in elderly patients. It is thus a pathology with a considerable socio-economic burden. Accurate diagnostic assessment including either echocardiographic or catheter-based pressure measurements is mandatory to quantify AS severity degree and to define treatment indication (30). Given the complex nature of the pathological patterns of aortic flow and WSS encountered in AS patients, a more detailed and multimodal diagnostic assessment is desirable and necessary to identify the best patient-individual treatment strategy and the benefits of patient-specific modelling of hemodynamics in that context are widely recognized (15, 21–24, 31).

In this study, we use CFD generated hemodynamic data to develop an artificial neural network (ANN) which computes clinically relevant hemodynamic parameters for AS patients. Using high resolution computed tomography (CT) image data, a large set of patient-specific hemodynamic CFD simulations is built based on a workflow developed earlier (22). Using these simulation results, an ANN is trained to predict pressure and WSS from patient specific geometry in a compact, centerline-based representation. Additionally, the centerline-based pressure and WSS is computed for a separate set of test cases using ANN and CFD to assess the ANN’s ability to compete with CFD simulations.

## 2. Materials and methods

### 2.1. Clinical data acquisition

Temporally resolved CT image data sets of 103 patients with AS who were treated in two different centers between February 2019 and October 2020 were retrospectively analyzed.

Inclusion criteria were the presence of aortic valve stenosis with indication for aortic valve replacement according to the European Society for Cardiology (ESC) guidelines and the interdisciplinary decision of the Heart Team and the availability of temporally resolved, high-resolution CT images for each patient. No further exclusion criteria were defined. The study was registered at ClinicalTrials.gov (NCT04600739) and was approved by the internal review board (EA2/174/19). Individual informed consent was waived due to the retrospective nature of this study. Patient’s characteristics at baseline are depicted in Table 1.

Computed tomography image data sets of the entire heart were acquired prior to the TAVI-procedure. The detailed protocol was previously published (32). An electrocardiogram-synchronized scan was conducted using either a wide area-detector volume CT scanner (Aquilion One Vision, Canon Medical 79 Systems, or Revolution CT, GE Healthcare) or a dual-source multi slice spiral CT scanner (Somatom Definition Flash, Siemens 78 Healthcare). Intravenous contrast medium was injected prior to each examination in order to improve image data contrast.

To allow the exact identification of the systolic phase with the widest aortic valve opening area, a multiphase data set, i.e., multiple scans at different time points, was reconstructed for each patient. All images were reconstructed with a soft-tissue convolution kernel and with the use of a dedicated noise reduction software. The spatial resolution used for segmentation was (0.39–0.648 mm) x (0.39–0.648 mm) in-plane resolution and (0.5–1 mm) slice thickness. The temporal resolution ranged from 70 ms to 140 ms.

Baseline echocardiographic evaluation results are additionally presented in Table 1. The echocardiographic data shows a wide range of aortic valve area (AVA) within the patient cohort, including cases with healthy AVA (>1 cm^2^), moderately (up to 0.7 cm^2^) and highly stenosed (<0.5 cm^2^) cases.

**Table 1:** Patient characteristics.

**Patient characteristic**	**Mean ± SD**	**Range**
**General patient information**		
Age [years]	82 ± 5	61–94
Height [cm]	168 ± 10	145–195
Weight [kg]	77 ± 19	35–135
**Echocardiographic assessment**		
TPG [mmHg]	61.9 ± 22.0	20–118
AVA [cm^2^]	0.74 ± 0.17	0.4–1.1
Stroke volume [ml]	52.2 ± 16.7	17–97
Ejection fraction [%]	57.3 ± 8.9	25–73

### 2.2. Image data segmentation

**Figure 1:**
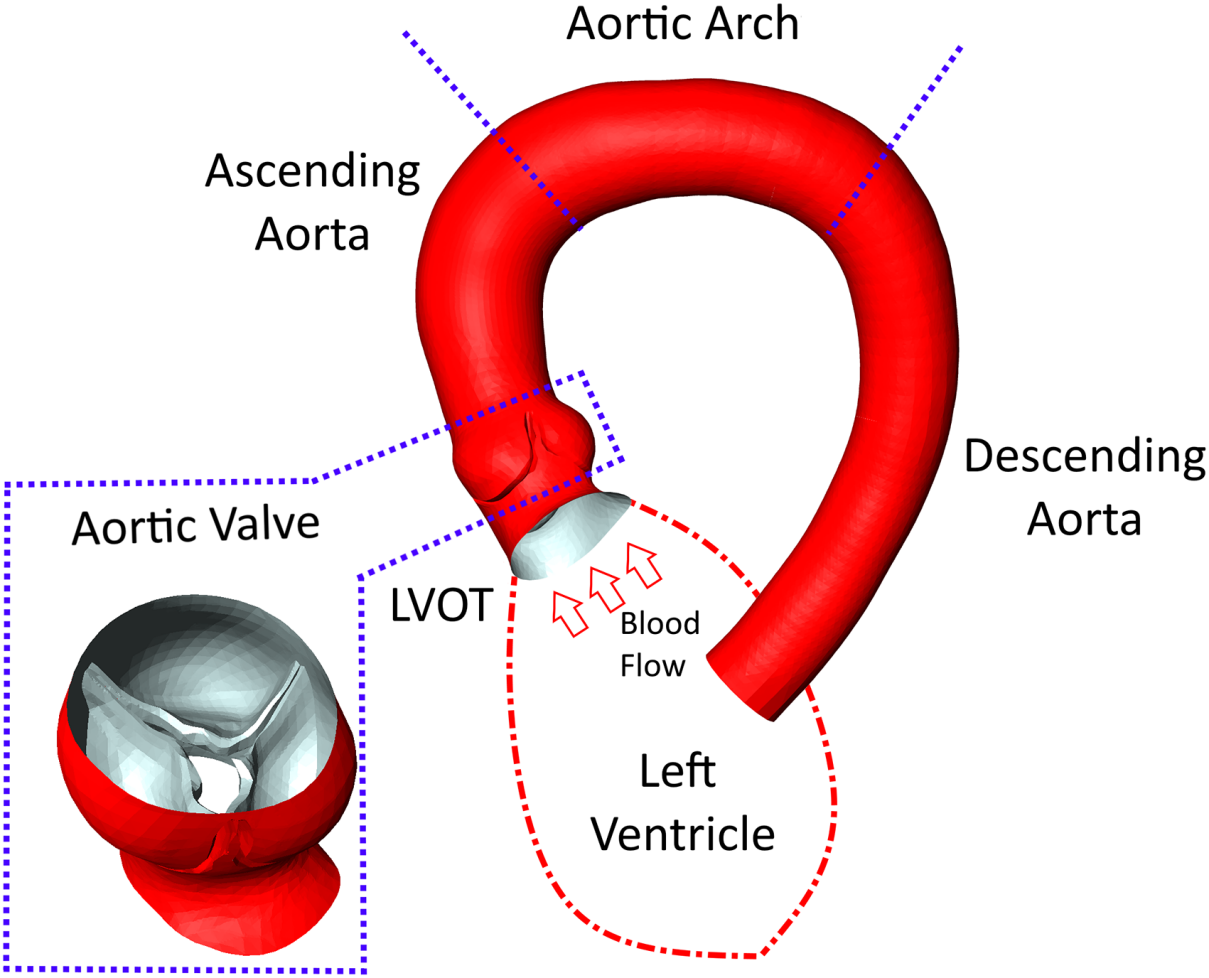
Surface model of the aorta and aortic valve of a typical case with severe aortic stenosis. Additionally, labels for the various parts of the aorta are provided as well as a schematic depiction of the left ventricle attached to the left-ventricular outflow tract (LVOT).

Based on the acquired CT data, the patient-specific surfaces of the aorta and aortic valve were segmented semi-automatically using a shape-constrained deformable model that was described earlier (32). Briefly, a parametric surface model of the aorta and aortic valve (33) is automatically adjusted to fit a given set of patient-specific CT images. Since the aortic valve is expected to be fully opened during the ejection phase simulated here, the valve is reconstructed in its fully-opened position using the respective CT images from the multiphasic dataset. An additional manual adjustment of the leaflet surfaces ensures an accurate representation of the aortic valve, which is critical for calculating transvalvular and aortic hemodynamics. Aorta and aortic valve surfaces are then combined into a single surface that defines the flow domain for the CFD simulation and the ANN input. The final model includes the left-ventricular outflow tract (LVOT), the open valve as seen at the time of peak systolic flow and the ascending aorta, aortic arch and descending aorta. Aortic branching vessels are not included in the model since their impact on hemodynamics in the valve region is considered negligible. Figure 1 shows a typical surface geometry with a labelling of the different parts (LVOT, valve, ascending, arch, descending aorta) as well as a close-up of the aortic valve.

### 2.3. Numerical simulation

Numerical flow simulations were performed using Siemens StarCCM+ v.15.04 (Siemens PLM, Plano, Texas). StarCCM+ uses the finite volume method to numerically solve the incompressible Navier–Stokes governing equations for the pressure and velocity fields. Given the high Reynolds number expected, a k-omega SST was used to account for turbulence. Blood was considered incompressible with a shear rate dependent viscosity based on the works of Abraham et al. (34) to account for the non-Newtonian behavior of blood (density = 1,050 kg/m^3^, zero/infinite-shear viscosity = 0.16/0.0035 Pa·s).

**Figure 2:**
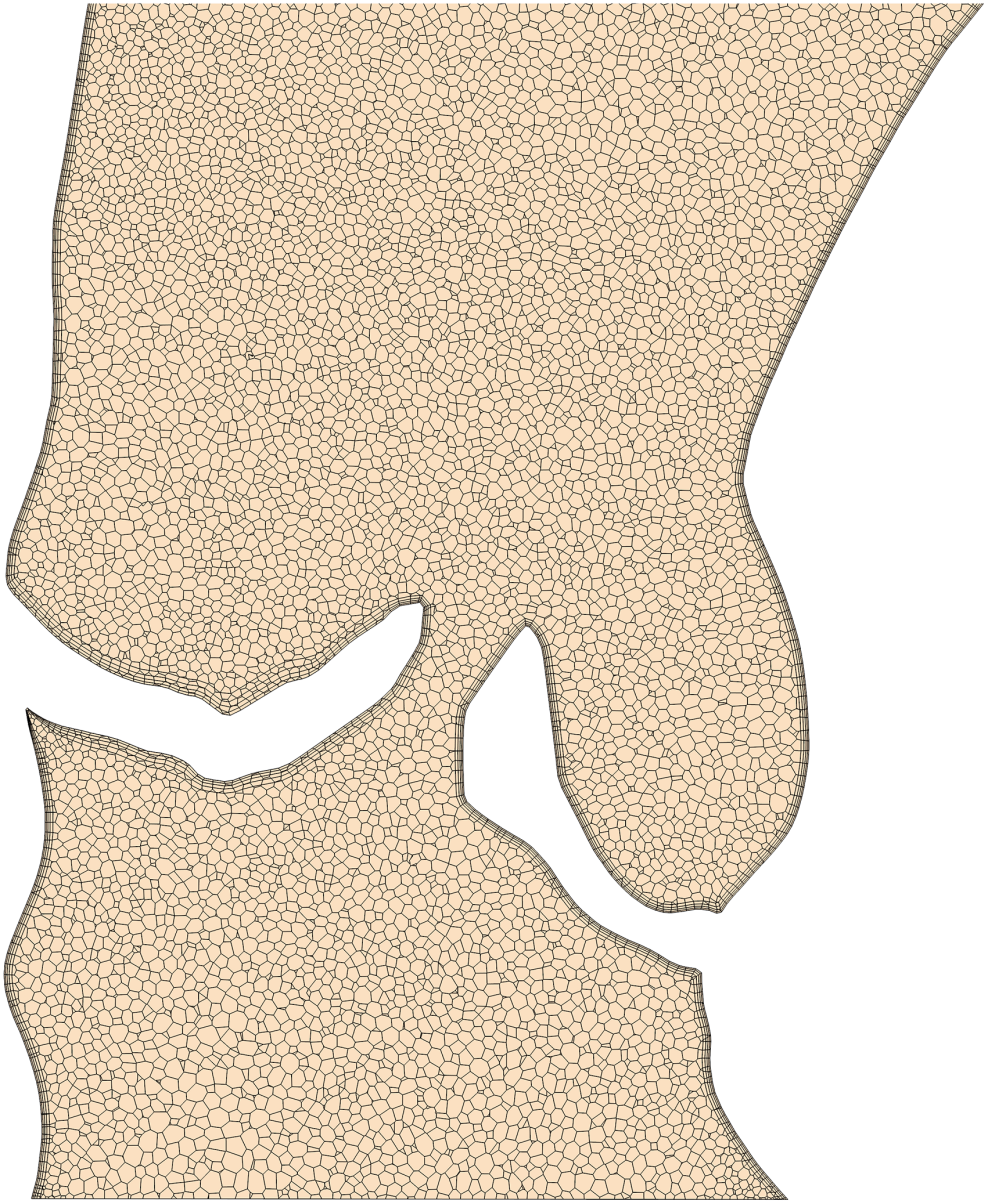
An example of the computational mesh structure in the aortic valve and ascending aorta used for the computational fluid dynamics simulations. The boundary layer structure used to resolve near wall flow can be seen around the valve and vessel walls.

Discretization of the flow domain was performed using StarCCM+‘s built in polyhedral meshing algorithm with five boundary layers to improve near wall flow resolution. Mesh size and structure is based on experience from similar studies made previously (22) for which mesh independency was analyzed. The analysis compared centerline-based pressure and WSS between four different mesh sizes ranging from very fine (10 million cells) to coarse (0.5 million cells) meshes. Medium sized meshes containing approximately 1.5–3 million cell/ 5–15 million nodes (depending on the size of the flow domain) yielded the best balance between accuracy and runtime showing a mean difference of 1–2 mmHg/2–4 Pa for pressure and WSS, respectively, compared to the reference solution. Figure 2 shows a longitudinal cross section of the mesh in the LVOT, aortic valve and ascending aorta.

Owed the complex nature of three-dimensional viscous fluid flow on the one hand and the limited amount of clinical data on the other, some simplifications regarding the numerical modelling process were necessary however. Simulations were therefore performed with steady flow boundary conditions at peak systolic flow rates, i.e., the maximum flow rate achieved during the ejection phase of the cardiac cycle. Furthermore, vessel wall and valve leaflets were considered rigid. Thus, the simulations model peak-systolic, instantaneous hemodynamics only, where the AS induced transvalvular pressure gradient (TPG) is expected to have the highest relevance. A transient solver (second order backward, 0.001 s time step) was used however to capture small-scaled unsteady flow effects such as flow separation and the valve jet mixture layer. The time step size of 1 ms ensures that the CFL-number remains at the order of one for a sufficient numerical stability and temporal resolution of the aforementioned unsteady effects.

Peak systolic flow rates were derived from left-ventricular volumetry (LVV) (32), which computes flow rate from the change of ventricular volume over time. However, multiphasic imagery suitable for LVV was available for only 67 of the 103 patients. For the remaining 36 patients, flow rates were drawn from a normal distribution whose mean and standard deviation were shifted based on case-specific AVA. This provided physiologically plausible flow rates with a certain degree of randomness, thus avoiding any deterministic relationships between geometry and flow rate which might harm the ANNs ability to generalize. Inlet boundary pressure was not prescribed since the pressure level has no influence on the solution of incompressible flow. However, pressure values were shifted to match an outlet pressure of 130 mmHg during post-processing for improved presentability. This value is based on the average of systolic pressure found in our patient cohort (Table 1).

**Figure 3:**
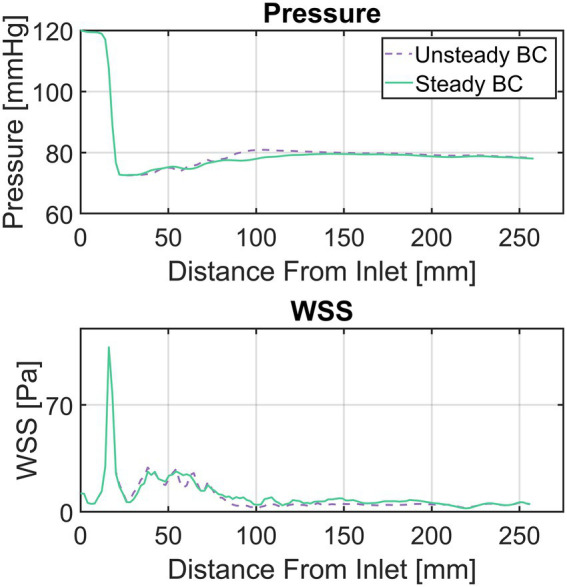
Comparison between a simulation with unsteady flow boundary conditions (BC) and a steady flow BC at peak systolic flow rate for a patient with a severe aortic stenosis. Top diagram compares pressure, bottom diagram wall-shear-stress (WSS). The flow curve for the ejection phase as well as the peak-systolic flow rate were derived from left-ventricular volumetry data.

Although the ability of the quasi-steady approach utilized here to accurately compute peak-systolic hemodynamics was investigated in earlier work (21, 22, 35), an additional analysis was performed for this study. Here, a flow rate curve was derived from LVV data for one patient with a severe stenosis and an unsteady simulation was performed containing the entire ejection phase. The centerline based values for pressure and WSS (as described in the next section) were compared between the unsteady and the quasi-steady simulation. The results of this comparison are shown in Figure 3 with centerline based pressure and WSS plotted for both simulations. The mean absolute and relative errors for pressure and WSS were found to be 0.7 mmHg/1.2% and 1.8 Pa/1.6% respectively, further supporting the validity of the peak-systolic simulation approach.

In addition to the baseline peak-systolic flow rates, simulations with varied flow rates were also performed to increase the amount of training data for the ANN. Flow rates were varied by ±25% of the respective base peak-systolic flow. This flow variation produces a desired substantial change in hemodynamics and corresponds with literature data on exercise-induced peak systolic flow change (36–39). This increased the total amount of simulations from 103 to 309.

### 2.4. Computational fluid dynamics post-processing

Although it is technically possible to use the three-dimensional data fields to train the ANN, a more compact representation of the simulation results becomes necessary considering the limited amount of training data available. This compact representation is constructed by locally averaging pressure and WSS along the aortic centerline using cross-section planes. This centerline-based representation substantially reduces input data dimensionality while retaining key spatial information. Moreover, this representation has shown to be more feasible for clinical use in our experience than three-dimensional data fields. Constructing the centerline-based representation of static pressure is performed in three steps:

- Generation of a discrete (2 mm point spacing) aortic centerline from the case-specific surface model.
- Creation of centerline-orthogonal vessel cross-sections at each centerline point.
- Calculating the average of static pressure on each cross-section and assigning the resulting value to the respective centerline point.

In the valve region, only the area bound by the valve leaflets is used to calculate cross-section averaged pressure.

**Figure 4:**
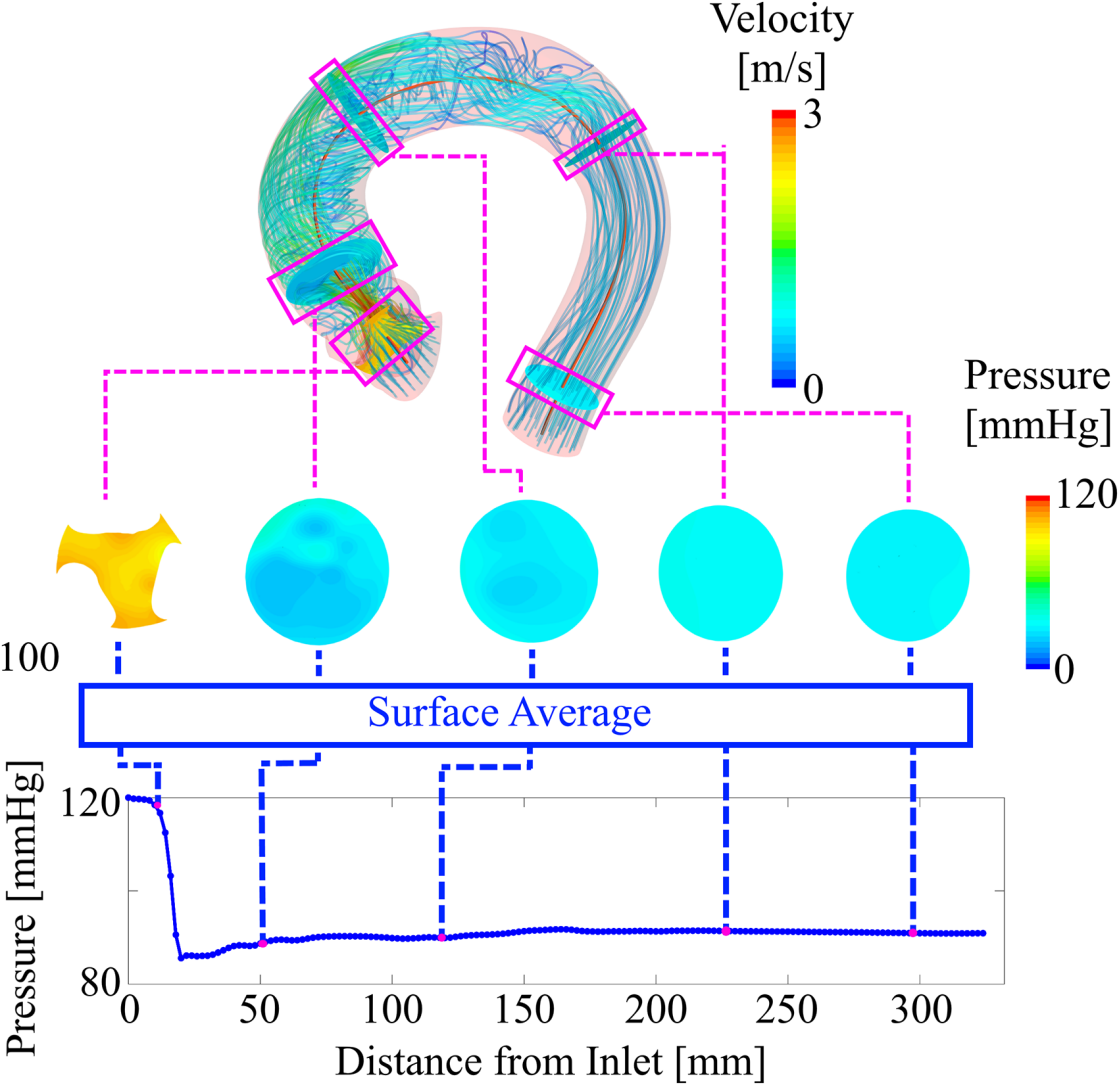
Deriving the compact representation (bottom plot) from the three-dimensional pressure and velocity field (top). Cross-section planes are defined along the vessel centerline. The average values of pressure on these planes are used to plot locally averaged pressure along the vessel centerline. Similarly, average values of WSS are computed from vessel sections between two adjacent cross-sections.

For WSS, the process is identical except that WSS is not averaged on the cross-sections planes but on vessel segments between two adjacent cross sections. For WSS in the valve region, only the inner leaflet surfaces are used to compute local WSS. Figure 4 illustrates the creation of the centerline-based representation from the CFD simulations.

### 2.5. Artificial neural network architecture

A recurrent neural network (RNN) is used to compute the hemodynamic parameters of pressure and WSS in the compact representation described above from a sequence of input features. These two hemodynamic parameters are chosen based on their clinical relevance. Static pressure derived TPG is the primary parameter for AS diagnostic and treatment (30) whereas aortic WSS is known to be a factor for aneurysm growth and other vessel degradation mechanisms (40–43).

A bi-directional long short-term memory (LSTM) RNN is used. This choice of architecture is well suited to work with the sequence-like data provided by the centerline-based representation and has proven successful in a similar study published earlier (28). The RNN is built using Matlab’s Deep Learning Toolbox R2020a (MathWorks, Natick, MA, United States).

**Figure 5:**
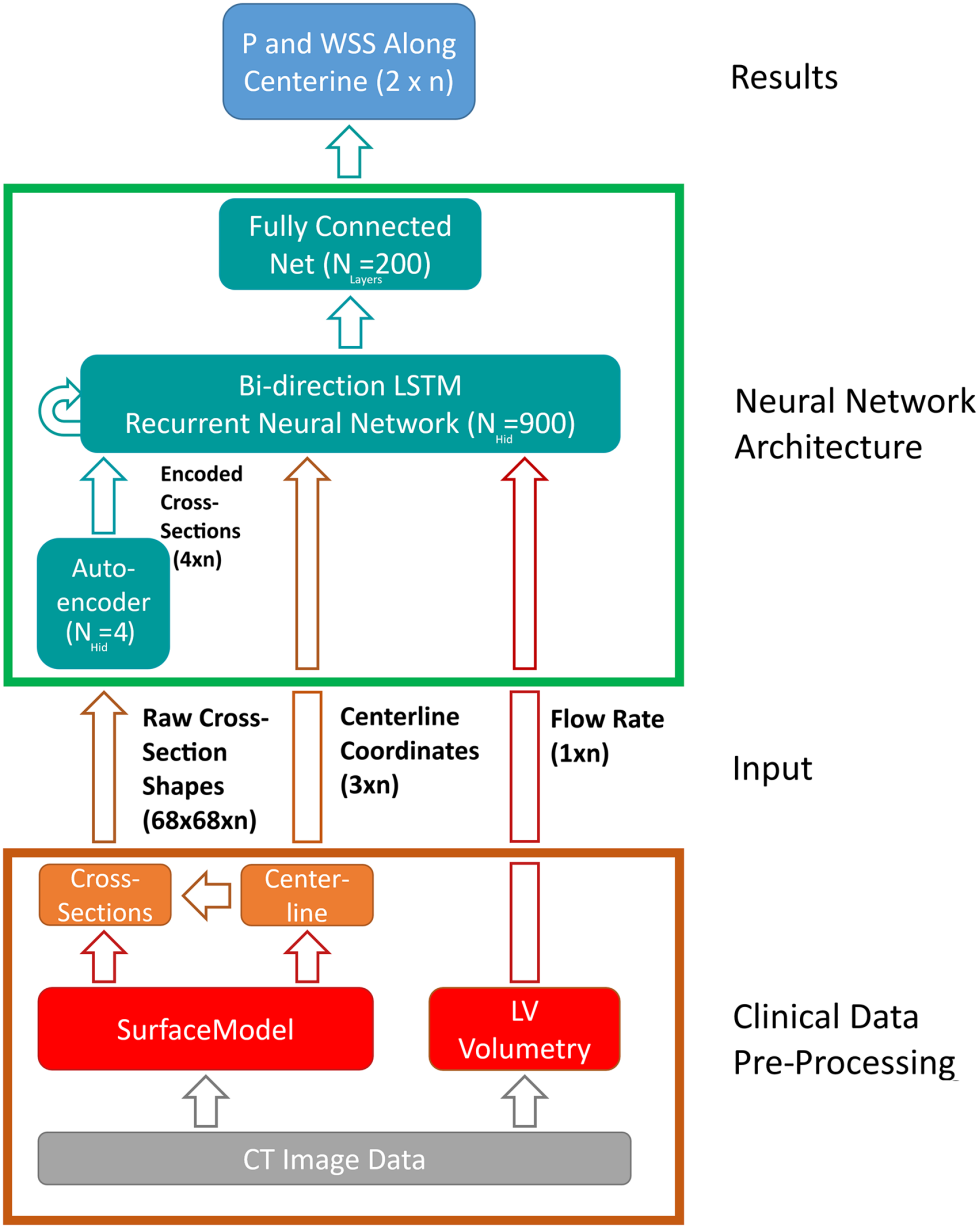
Schematic depiction of the artificial neural network (ANN) based workflow for computing pressure and wall-shear-stress (WSS) along the vessel centerline. Clinical data pre-processing includes the extraction of the vessel surface model as well as peak systolic flow rate from multiphasic computed tomography (CT) image data. Vessel centerline and cross-section shapes are then derived from the surface model and passed to the ANN directly (centerline-coordinates) and through an auto encoder, respectively. A long-short-term-memory (LSTM) recurrent neural network computes the flow states at each centerline point based on centerline coordinates, encoded cross-sections and flow rate. Lastly, a fully connected layer derives pressure and wall-shear-stress along vessel centerline from the flow states provided by the LSTM. Hidden state and input/output sizes are also provided in the schematic.

The LSTM uses a sequence of input features derived from the case-specific surface model and flow rate and outputs a sequence of state vectors. From these state vectors, a fully connected layer computes the values of pressure and WSS along the vessel centerline. Figure 5 illustrates the various components of the network as well as the data flow through the components. Both input and output sequences are centerline-based, meaning their values are defined at their respective centerline points. The following input features are used:

- Centerline point coordinates (n × 3).
- Cross-section area (n × 1).
- Cross-section area normalized flow rate (n × 1).
- m-dimensional encoded cross-section shape (n × m).

Where n denotes the number of centerline points for a given case (usually 110–130, depending on the length of the aorta).

The last feature is provided by an auto encoder (AE) which is trained separately. Using the AE, information on cross-section shape can be passed to the LSTM using only a few parameters of size m instead of complex polygons or triangulations. This information is deemed necessary since valve geometry varies substantially between individual cases and using cross-section area alone might not suffice to account for this geometrical variation.

The AE used here is part of Matlab’s Deep Learning toolbox and consists of a single-layer neural network (the encoder) that maps an input of size n to a hidden state of size m. From this hidden state, a second neural network (the decoder) reconstruct the original input data. The error between original and reconstructed input allows to define a cost function which is used to extract information critical to ANN accuracy from the original input (in this case cross-section shape), thereby allowing to substantially reduce input dimensionality without compromising accuracy. The size of the encoded state m (i.e., the amount of ‘essential’ information contained in the raw cross-section shapes) is determined during hyperparameter optimization. Further reference on the AE used in this study can be found in the respective Matlab documentation (44).

### 2.6. Artificial neural network training and hyperparameter optimization

A total of 309 simulation results were available for training and validation. This dataset was reduced to 267 cases based on a maximum pressure drop (MPD), defined as the difference between inlet pressure and the lowest pressure found within the vessel, of 120 mmHg. This removed cases with an unphysiologically high pressure drop caused by either an excessive flow rate (+25% or modelled flow), an underestimation of valve area (i.e., segmentation errors) or a combination of both. The threshold of 120 mmHg is based on clinical guidelines, where TPG of 60 mmHg is already considered severe (30). Thus, an MPD in excess of twice this value is believed to be beyond what is found in the vast majority of AS patients. This is further supported by the echocardiographic TPG data obtained for this cohort (Table 1). Although this retains cases with a TPG that is well above any value considered critical for an intervention, it is believed that retaining this data is beneficial for the ANN’s accuracy in the clinically relevant range.

The remaining 267 cases were split into 11 datasets, one for testing and the other 10 for training and validation/optimization. Stratification was achieved by ensuring that each subset covers the whole range of allowable MPD (approx. 0–120 mmHg). Moreover, individual geometries were not distributed across subsets, meaning that a particular case will have all of its flow variations (baseline ±25%) within one subset. Thus, it is ensured that validation/test data remains fully unknown during training/testing. The test subset contained 23 cases, leaving 244 cases for training and hyperparameter optimization.

Network training was performed using adaptive moment estimation, a stochastic gradient descend algorithm that aims at minimizing the loss function (i.e., the difference between desired and actual network output). Hyperparameter optimization was performed using grid search. For each configuration, a 10-fold cross validation was performed. The configuration with the highest average accuracy was chosen as the final model. The following hyperparameters were considered (optimum values in brackets):

- AE input size (68 × 68).
- AE hidden size (4).
- LSTM hidden size (900).
- Fully connected layer size (200).

ANN robustness and explainability was analyzed using a local feature perturbation approach used earlier (28). This method locally changes the input feature sequences around a given centerline point by ± one standard deviation and measures the output RMSE between the altered and original input.

### 2.7. Artificial neural network accuracy assessment

ANN accuracy was assessed by computing the root-mean-square-error (RMSE) between CFD- and ANN-based pressure/WSS on the test dataset. The pressure RMSE for a specific case is defined as:


(1) $$RMSE_{P}=\sqrt{\frac{\sum_{i=1}^{N}{\left(P_{i}{}_{ANN}-P_{i}{}_{CFD}\right)}^{2}}{N}}\;$$

Where $P_{i}{}_{ANN}$ and $P_{i}{}_{CFD}$ are the ANN/CFD-based values of pressure at the *i-th* centerline point and N is the number of centerline points.

RMSEs for pressure and WSS were furthermore normalized to provide a relative measure of accuracy. Pressure RMSE was normalized with CFD-based MPD whereas WSS was normalized with the maximum WSS observed in the respective CFD result.


(2) $$NRMSE_{P}=\sqrt{\frac{\sum_{i=1}^{N}{\left(\frac{P_{i}{}_{ANN}-P_{i}{}_{CFD}}{P_{1}{}_{CFD}-\inf\left\{P_{1}{}_{CFD},\ldots ,P_{N}{}_{CFD}\right\}}\right)}^{2}}{N}}$$

(3) $$NRMSE_{WSS}=\sqrt{\frac{\sum_{i=1}^{N}{\left(\frac{WSS_{i}{}_{ANN}-WSS_{i}{}_{CFD}}{\sup\left\{WSS_{1}{}_{CFD},\ldots ,WSS_{N}{}_{CFD}\right\}}\right)}^{2}}{N}}$$

Additionally, a test for statistical equivalence is performed using a two one-sided tests (TOST) procedure. TOST based equivalence tests decide whether two different measuring methods applied on a set of subjects can be considered statistically equivalent within some bounds $\varepsilon_{L}$ and $\varepsilon_{U}$. In this study, the ANN- and CFD-based computation of TPG, which is the primary clinical parameters for AS assessment, are tested for equivalence as follows:

Let $\mu_{CFD}$ and $\mu_{ANN}$ be the median values of the CFD and ANN-based TPG values. Then the median difference:


(4) $$\Theta =\mu_{CFD}-\mu_{ANN}$$

is hypothesized to be outside of a reference value $\Theta_{0}$ ± an equivalence margin ε using two separate null-hypotheses as:


(5) $$H_{1}^{0}:\Theta <\Theta_{0}-\varepsilon$$

(6) $$H_{2}^{0}:\Theta >\Theta_{0}+\varepsilon$$

Hypotheses (5) and (6) are tested using a signed rank test, since the TPG values are not normally distributed in our data. If both (5) and (6) can be rejected at some significance level α, it follows that both methods are equivalent in computing TPG within the bounds provided by ε (45, 46).

**Figure 6:**
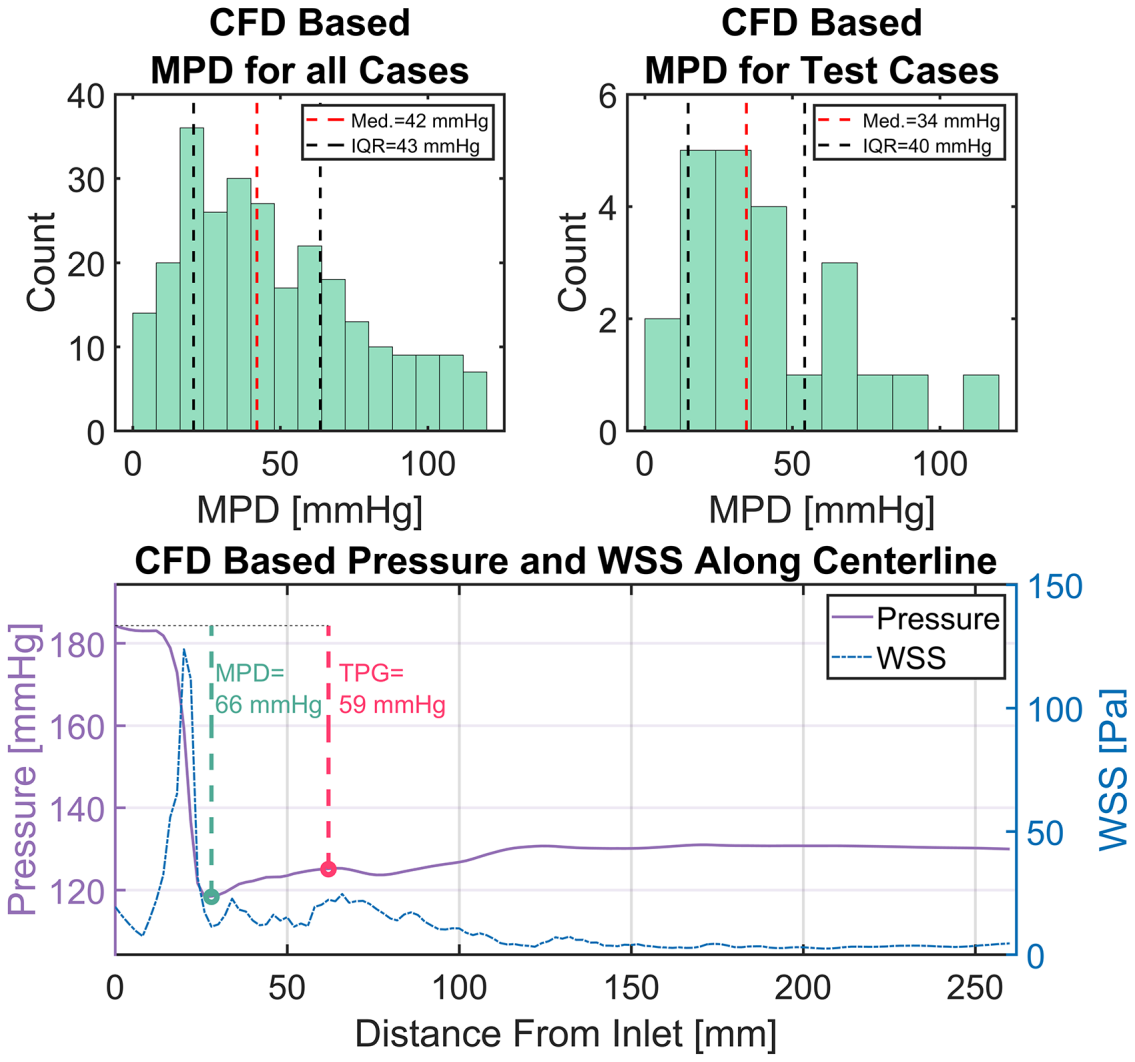
Top left: Distribution of maximum pressure drop (MPD) within the entire simulation data. Top right: Distribution of MPD within the set of test cases. Bottom: An exemplary depiction of CFD-based pressure and wall-shear-stress (WSS) in the compact representation (i.e., average along centerline). Additionally, the measurement points for transvalvular pressure gradient (TPG, red) and MPD (green) are shown with their respective values.

For this study, an equivalence margin ε of ±5 mmHg is used which corresponds to the accuracy of current clinical catheter-based pressure measurements (47). TPG is defined as the difference between inlet pressure and the pressure at the point of highest pressure recovery downstream of the aortic valve, exemplary depicted in Figure 6 (bottom).

## 3. Results

### 3.1. Computational fluid dynamics results

A total of 309 simulations were performed using 103 patient-specific geometries (baseline flow ±25%). 42 simulations were excluded based on an MPD of 120 mmHg as mentioned previously, leaving 267 simulations results. Figure 6 (top left) shows a histogram of the MPD distribution found in the simulation results. MPDs were not normally distributed with low to moderate MPD values (<40 mmHg) dominating the distribution. Median of MPD is 42 mmHg with an inter-quartile range (IQR) of 43 mmHg. The MPD distribution within the set test cases (Figure 6, top right) is equal to that of all cases (Wilcoxon rank sum test, *p* < 0.05) with a median of 34 mmHg and an IQR of 40 mmHg. The available training data is therefore biased towards moderate MPD values/stenosis severity. However, low and high MPD values appear sufficiently represented.

Figure 6 (bottom) provides an example of a typical case with a high pressure drop. The plot shows the simulation results in the compact representation described earlier, depicting locally averaged values for pressure and WSS along the vessel centerline. Additionally, the points defining MPD and TPG are shown with a green and red circle, respectively.

The characteristic rapid pressure drop and increase of WSS in the valve region can be seen as well as a slight pressure recovery in the ascending aorta along with a reduction of WSS. Moreover, a slight fluctuation of WSS with local maxima in the ascending aorta are observed, which is a result of secondary flow structures in that region generated by the valve jet.

### 3.2. Artificial neural network explainability and robustness

The explainability and robustness analysis revealed that the network is most sensitive to perturbations in the valve region while changes to the input downstream of the aortic valve have little effect on output (RMSE <1 mmHg). Moreover, the input parameters of flow and encoded cross-section shape appear to have the most influence on the hemodynamic results provided by the ANN. Reducing cross-section area/shape and/or increasing flow-rate results in an increase of TPG and *vice-versa* (RMSE 10–20 mmHg). These finding are in line with the fluid dynamic behavior expected for such a configuration, i.e., pressure drop over a local constriction as a function orifice area and flow velocity.

### 3.3. Artificial neural network accuracy

Median of absolute pressure RMSE for all 23 test cases was 2.0 mmHg with an IQR of 3.2 mmHg. Absolute WSS RMSE was normally distributed (Lilliefors test at *p* < 0.05) with a mean of 5.3 ± 3.9 Pa. Normalized pressure and WSS RMSE were not normally distributed with a normalized pressure RMSE median of 6.0% (IQR 5.7%) and a normalized WSS RMSE median of 4.9% (IQR 1.5%). A significant (*p* < 0.001) correlation between case-specific RMSE and MPD/peak WSS was observed, with correlations of 0.53 and 0.71, respectively. Neither geometric parameters such as AVA or aortic diameter/length nor the type of flow boundary condition (i.e., LVV based or modelled flow rate) were found to correlate with ANN accuracy.

**Figure 7:**
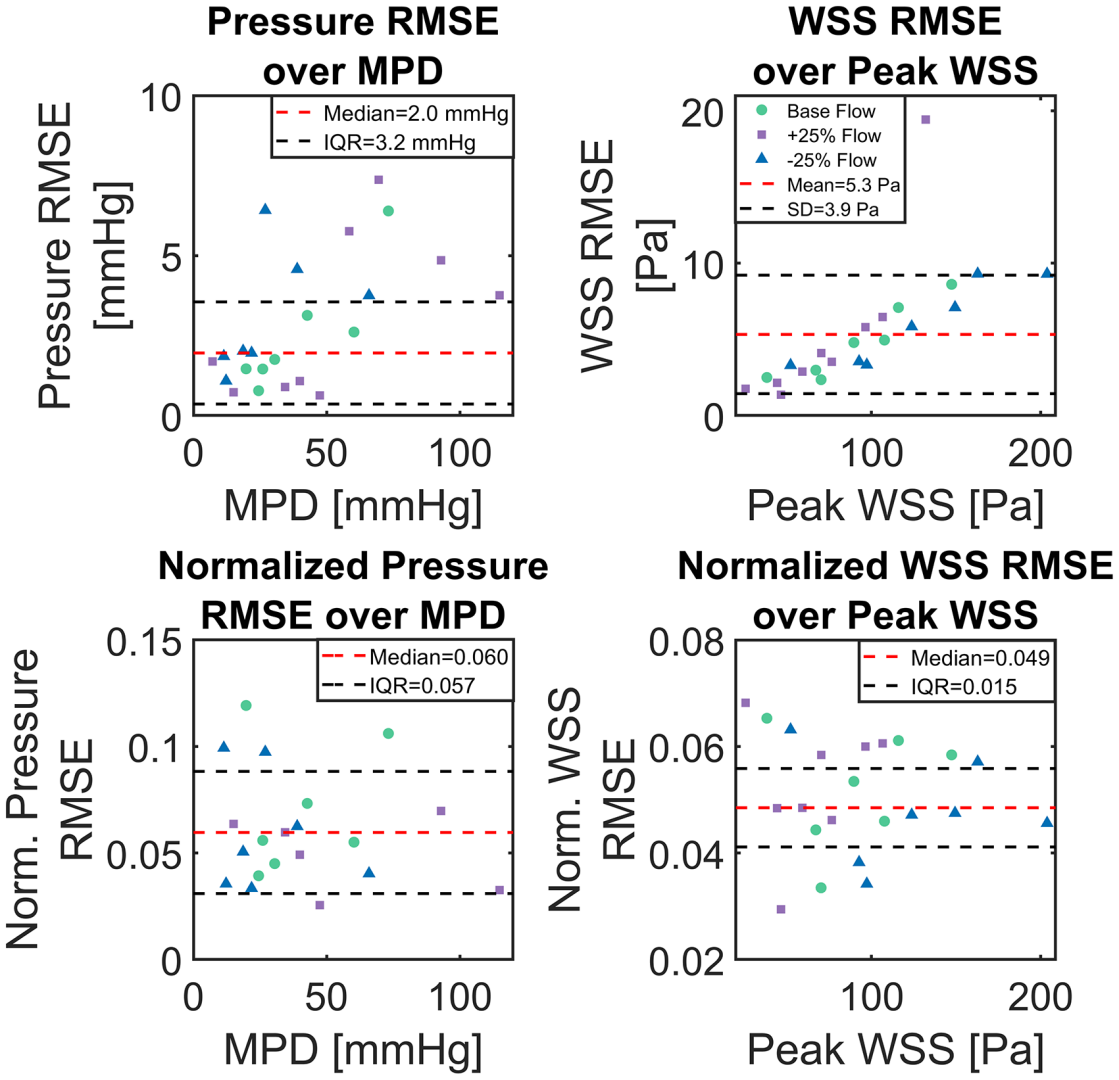
Scatter plots of absolute and normalized root-mean-square-errors (RMSE), calculated between computational fluid dynamics (CFD) simulations and artificial neural network (ANN)-based results for the set of 23 test cases. Top shows absolute pressure and wall-shear-stress (WSS) RMSE over CFD computed maximum pressure drop and maximum WSS, respectively. Bottom shows pressure and WSS RMSE normalized with TPG and maximum WSS, respectively.

Figure 7 shows scatter-plots of absolute (top) and relative (bottom) pressure and WSS RMSE over MPD and peak WSS for the 23 test cases. Data points in Figure 7 are color and shape coded to discriminate cases with baseline flow from those with reduced/increased flow. The absolute RMSE plots visually confirm the tendency of higher absolute RMSE with higher MPD. Moreover, an increased dispersion of absolute pressure RMSE can be seen at higher MPD whereas absolute WSS RMSE increases along a narrow band. A sole outlier with a comparably high WSS RMSE of 19.4 Pa is present though. A tendency of either baseline or +/− 25% flow to produce particularly large RMSE for pressure or WSS could not be observed.

**Figure 8:**
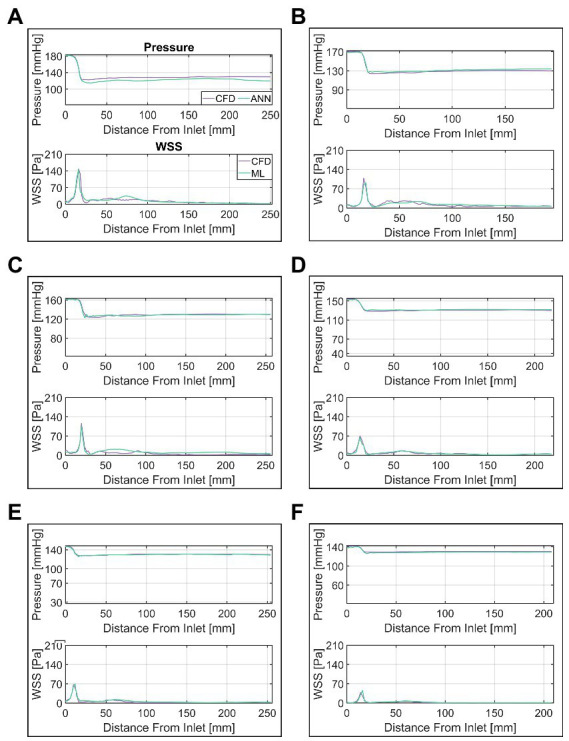
Comparison of ANN and CFD-based pressure and wall-shear-stress (WSS) along vessel centerline. Cases (**A–F)** are ordered by descending transvalvular-pressure-gradient.

A more detailed presentation of the ANN’s performance is provided in Figure 8. In this figure, a selection of six cases (A through F) of various MPD/TPG levels are shown with their respective CFD and ANN-based pressure/WSS along vessel centerline. ANN and CFD computed pressure match closely throughout the whole vessel except for case A, where the pressure drop in the valve region is overestimated by approximately 8 mmHg. This error propagates further downstream resulting in an overall discrepancy between ANN- and CFD-based pressure. Moreover, a slight fluctuation of pressure can be seen in cases B, C and E at the point of lowest pressure which is not present in the CFD data.

WSS appears to match similarly well between CFD and ANN, however, notable differences occur in the ascending aorta. In that region, the ANN either overestimates WSS (case C) or fails to capture local fluctuations (case B) or a combination of both (case A). WSS in the valve region on the other hand matches well between ANN and CFD.

Finally, the TOST for TPG measurement equivalency between CFD (median 32 mmHg, IQR 37 mmHg) and ANN (median 34 mmHg, IQR 33 mmHg) resulted in both methods being equivalent within the equivalency bounds of ±5 mmHg (*p* < 0.001).

## 4. Discussion

In this pilot study, we presented a deep ANN computing spatially resolved, compact hemodynamics in the aorta and aortic valve of AS patients as an alternative to traditional numerical modelling. The network was trained using 244 patient-specific CFD simulations of the aorta and aortic valve while 23 simulation were used for the evaluation of the ANN’s performance. Modelling hemodynamics using AI has been undertaken by several studies earlier (27, 28, 48, 49). However, this kind of application is still rather underrepresented in the medical field (50–52), although it appears worthwhile exploring given the potential benefits outlined in the introduction.

The comparison between ANN- and CFD-based pressure computations provided in the results section showed good agreement between both methods with differences being at the order of a few mmHg. Considering that clinically relevant TPG values are at the order of 20 mmHg and upwards (30), the observed pressure error can be considered rather low. For the computation of TPG, both methods can even be considered equivalent within current clinical pressure measurement margins.

Assessing the accuracy of ANN-based WSS computation is more difficult, since no current clinical guidelines for WSS measurement or evaluation exist. Thus, the mean error of approx. 5 Pa is difficult to put into a meaningful clinical perspective. However, judging by the normalized accuracy, WSS and pressure computations are similarly accurate, although some test cases showed substantial errors in the ascending aorta. Another interesting finding is that ANN accuracy does not suffer from altering the flow rate given any particular geometry. This may provide an important capability for clinical use, as outlined further below.

All in all, an ANN-based modelling of aortic hemodynamics of AS patients appears to be a viable alternative to CFD. Considering the enormously lower computational time required (hours for CFD vs. seconds for ANN), ANN-based methods may facilitate the translation of hemodynamic modelling into clinical practice, something that CFD-based methods struggle with, despite their potential (15, 21–23, 53).

### 4.1. Potential clinical application

In the context of the use case of AS discussed here, such an ANN could be embedded into a CT or magnetic resonance imaging (MRI) scanner, expanding the diagnostic capability of the device to provide treatment-critical hemodynamic information (e.g., pressure, TPG) along with image data in real-time. This could in turn reduce the requirement for invasive diagnostics of TPG and thus reduce costs and patient risk. Furthermore, the change in TPG under increased cardiac output (i.e., flow) could be simulated for borderline-symptomatic patients, thus reducing or even replacing the current practice of measuring pressure under drug or exercise induced stress for such patients (54, 55). Finally, the ANN presented here can be expanded to model aortic hemodynamics for bicuspid aortic valves, prosthetic valves such as TAVI, biological or mechanical valve replacement, thereby providing treatment planning and hemodynamic outcome prediction capabilities (21, 22).

### 4.2. Error analysis and potential improvement

Although the results are promising, the work presented here does not fully provide the potential capabilities outlined above yet and several factors need to be considered for a successful clinical translation. First of all, the ANN and CFD-based results show notable differences for cases with high MPD/TPG. Especially WSS appears to diverge substantially between the two methods in the ascending aorta, as seen in Figure 8. These errors indicate that the ANN does not fully capture the relationship between geometry, flow rate and pressure/WSS distribution inside the vessel.

For both pressure and WSS, an increase in ANN pressure and WSS RMSE was observed with an increase of MPD and max WSS, respectively. This was more pronounced for WSS, where maximum WSS and WSS RMSE where notably correlated. This suggests that the overall error can be viewed as a combination of a systematic error which depends on some parameter/feature and a random error. The systematic error may be explained by the fact that with higher MPD/maximum WSS, the ANN struggles to capture the resulting high spatial variation of these parameters along the centerline since the distance between the centerline points is fixed to 2 mm. Moreover, the loss of spatial information from the cross-section averaging process has a greater impact on cases with a severe stenosis and thus high MPD/maximum WSS. For such cases, the pressure/WSS on a single cross section plane may vary by up to 20 mmHg/50 Pa, which appears mostly in the region where the valve jet impinges the vessel wall. The cases displayed in Figures 8A–C confirm this, since the largest differences ANN and CFD-based WSS are seen downstream of the valve at the end of the ascending aorta (50–75 mm downstream). For cases with a less severe stenosis and thus weaker valve-jet, the differences are far lower making the cross-section averaged representation more true to the actual distribution of pressure and WSS.

In this context, the correlation between RMSE and MPD/max WSS may be seen as an indicator which of the two error sources, constant or random, dominates the overall error. Hence, the random error appears more influential for pressure RMSE than it is for WSS RMSE, where it supposedly accounts for half of the variance in the WSS RMSE (R^2^ of 0.5). This appears reasonable given the fact that the local variation of WSS across the vessel wall is higher than the local variation of pressure on the cross sections (with respect to the overall level of pressure/WSS). The random error, on the other hand, is likely the result of uncertainties in the input data, as will be discussed further below. All in all, the differences observed between CFD and ANN based solutions can be attributed to the following factors:

1. Uncertainty in the input data.
2. Random numerical errors in the training data (i.e., CFD).
3. Comparably low amount of training data/biased training data.
4. Input data pre-processing.

The first two factors mostly contribute to the random error described earlier. The uncertainty in the input data for instance, which results from inconsistencies in the definitions of the cross-sections/vessel segments in the valve region, may omit pressure/WSS data in some cases, leading to an erroneous input and thus result. The random numerical errors are the result of the discretization error inherent to the CFD method on the one hand and the fact that the pressure and velocity fields fluctuate slightly during runtime on the other. These are at the order of 1–2 mmHg based on the mesh independency study as well as the convergence behavior observed in our simulations.

The last two points are likely to influence both the systematic and the random error. More training data would provide a denser sampling of the parameter space (both geometric and hemodynamic parameters) and allow for more sophisticated inputs to be used, thus potentially reducing the random error. Moreover, additional cases in the high MPD range would remove the bias in the training data which could be another factor contributing to the systematic error besides the ones discussed earlier.

The pre-processing of input data plays a key role in how information is passed to the network. Currently, the raw simulation as well as the shape data are substantially reduced using the centerline-based representation. This was deemed necessary to efficiently utilize the clinical data available for this study. However, it creates the aforementioned problems of spatial discretization as well as the mismatch between average and actual pressure/WSS distributions. Furthermore, the auto encoder-based reduction of the cross section shape dimensionality is also likely to introduce errors into the input data and hence the results. Increasing input data dimensionality with a denser centerline sampling, a larger AE hidden-state size and more distribution parameters for pressure and WSS are likely to reduce both the systematic error as well as the random error through input data uncertainty. However, the possibility of providing additional input data is closely tied to clinical data availability.

Finally, the choice of ANN architecture itself is worth evaluating, although the question of optimal ANN architecture cannot be definitely answered within the scope of this study. While the choice of architecture used here appears reasonable given the underlying physical modelling problem, it is not necessarily the best one. Other ANN types such as convolutional neural networks, which are highly successful in working with image data, may produce even better results depending on available input data and required output. Evaluating different types of architectures should therefore be another key aspect of future studies.

### 4.3. Limitations

An important concern regarding the translatability of the ANN into clinical practice is the underlying CFD method, on which the ANN is trained. Since the ANN can only be as good as the training data it uses (i.e., the CFD data), it must be ensured that the CFD-based hemodynamic modelling is accurate and validated against clinical data. Although CFD simulations of aortic flow in AS patients using the setup employed here showed good agreement with clinical data in earlier studies (15, 21, 22, 32), a thorough validation under controlled conditions is necessary. This was not possible in this study since the CT imaging on which the CFD is based and echocardiographic measurements were performed on different appointments. This potentially introduces a number of unknown errors. In addition, echocardiographic TPG measurements are suspected to systematically overestimate TPG (56) making the clinical TPG data available here unsuitable for validation purposes. The above however does not necessarily impact the potential of an ANN to substitute CFD modelling, since CFD simulation inaccuracies often result from inaccurate input data (segmentation, flow boundary conditions) rather than from a lack of modelling complexity.

The steady-state, peak systolic computational model employed in this study is another limitation worth mentioning. This simplification was deemed necessary to find a good balance between modelling complexity and clinical relevance for this initial work. And while this approach may already provide clinically relevant data (15, 21, 35, 57), obtaining hemodynamic information from a whole cardiac cycle is certainly desirable. In particular, the time averaged TPG, which is another important factor in AS diagnosis and treatment decision (30), cannot be calculated using the quasi steady-state approach used here. Other clinically relevant hemodynamic parameters such as time-averaged WSS and the oscillatory shear index, which are hypothesized to influence aortic valve and aortic wall degeneration (58–61), also fall outside of the current capabilities of the ANN. Therefore, extending the CFD and the ANN to model unsteady hemodynamics is necessary to fully evaluate the potential of ANN-based hemodynamic modelling.

Finally, an important aspect necessary to realize an application of an ANN-based hemodynamic computation as outlined earlier in the section is the pre-processing of clinical input data. Although the ANN presented here partially uses image data for the input, this image data is not raw CT or MRI data and all input sequences are derived from a segmented surface of the aorta and valve. This surface is currently the result of a lengthy semi-manual segmentation of CT data, which negates the ANN’s primary benefits of low computational times and user interaction. Therefore, a fast segmentation algorithm capable of automatically extracting aortic centerline, aortic- and valve-surfaces from cardiac CT or MRI is necessary. The development of such algorithms is ongoing and several promising methods exist, however, automatic segmentation of cardiac image data remains a challenging task (62).

## 5. Conclusion

The ANN-based computation of pressure and WSS in patients with AS is a viable alternative to time- and resource-intensive CFD-based modelling. Requiring very little computational power or user interaction, ANN-based hemodynamic modelling can become a key part in the integration of hemodynamic modelling into clinical practice, thereby expanding diagnostic capabilities, reducing costs and improving outcome. However, in its current state, the ANN presented here needs further improvement to be suitable for a clinical application. Apart from improving the ANN’s accuracy as outlined in section 4.2, further work is required to validate ANN accuracy against clinical data as well as provide an automatic image segmentation tool in order to derive a hemodynamic outcome from raw image data in a clinical setting. Nevertheless, the suitability of ML-based methods to perform hemodynamic modelling has been demonstrated.

## Data availability statement

The datasets presented in this study can be found in online repositories. The names of the repository/repositories and accession number(s) can be found at: https://figshare.com, DOI: 10.6084/m9.figshare.19478117.

## Ethics statement

The study was registered at ClinicalTrials.gov (NCT04600739) and was approved by the internal review board (EA2/174/19). Written informed consent for participation was not required for this study in accordance with national legislation and the institutional requirements.

## Author contributions

PY and BF: data curation. PY and LG: formal analysis. LG, TK, and MS: funding acquisition. PY, LG, and MS: investigation and writing-original draft. PY: methodology and visualization. MS and LG: supervision. All: writing-review and editing, conceptualization, and contributed to the article and approved the submitted version.

## Funding

The work presented here is largely built on the “ArtiCardio” project, which was funded by the German Federal Ministry of Education and Research (BMBF), grant number 13GW0208B. MS is a participant in the BIH Charité Digital Clinician Scientist Program funded by the Charité – Universitätsmedizin Berlin, and Berlin Institute of Health at Charité (BIH). Additional funding was provided through professor Goubergrits by the Einstein Center Digital Future (ECDF). Finally, we would like to acknowledge financial support from the Open Access Publication Fund of Charité – Universitaetsmedizin Berlin and the German Research Foundation (DFG).

## Conflict of interest

The authors declare that the research was conducted in the absence of any commercial or financial relationships that could be construed as a potential conflict of interest.

## Publisher’s note

All claims expressed in this article are solely those of the authors and do not necessarily represent those of their affiliated organizations, or those of the publisher, the editors and the reviewers. Any product that may be evaluated in this article, or claim that may be made by its manufacturer, is not guaranteed or endorsed by the publisher.
